# Diabetes and the Brain: Oxidative Stress, Inflammation, and Autophagy

**DOI:** 10.1155/2014/102158

**Published:** 2014-08-24

**Authors:** María Muriach, Miguel Flores-Bellver, Francisco J. Romero, Jorge M. Barcia

**Affiliations:** ^1^Facultad de Medicina y Odontología, Universidad Católica de Valencia, Calle Quevedo 2, 46001 Valencia, Spain; ^2^Facultad de Ciencias de la Salud, Universitat Jaume I, 12071 Castellón, Spain

## Abstract

Diabetes mellitus is a common metabolic disorder associated with chronic complications including a state of mild to moderate cognitive impairment, in particular psychomotor slowing and reduced mental flexibility, not attributable to other causes, and shares many symptoms that are best described as accelerated brain ageing. A common theory for aging and for the pathogenesis of this cerebral dysfunctioning in diabetes relates cell death to oxidative stress in strong association to inflammation, and in fact nuclear factor *κ*B (NF*κ*B), a master regulator of inflammation and also a sensor of oxidative stress, has a strategic position at the crossroad between oxidative stress and inflammation. Moreover, metabolic inflammation is, in turn, related to the induction of various intracellular stresses such as mitochondrial oxidative stress, endoplasmic reticulum (ER) stress, and autophagy defect. In parallel, blockade of autophagy can relate to proinflammatory signaling via oxidative stress pathway and NF*κ*B-mediated inflammation.

## 1. Introduction

Diabetes mellitus is a common metabolic disorder which is associated with chronic complications such as nephropathy, angiopathy, retinopathy, and peripheral neuropathy. However, as early as 1922 it was recognised that diabetes also can lead to cognitive dysfunction [1]. Since then, studies in experimental models and in patients observed alterations in neurotransmission, electrophysiological and structural abnormalities, and neurobehavioral alterations, in particular cognitive dysfunction and increased risk of depression [2]. Moreover, the observed cerebral manifestations of diabetes appear to develop insidiously, largely independent of diabetes-associated acute metabolic and vascular disturbances (such as severe hypo- and hyperglycemic episodes and stroke). Although the magnitude of these cognitive deficits appears to be mild to moderate, they can significantly hamper daily functioning, adversely affecting quality of life [3].

In spite of this, the concept of central neuropathy has been controversial for more than 80 years now, but while trying to describe cognitive impairment in diabetes as a complication of the disease, the term “diabetic encephalopathy” was introduced in 1950 [4]. However, this term “encephalopathy” has not been widely accepted, probably among other reasons, because it does not seem to match with the mild cognitive problems usually seen in (nondemented) diabetic patients. More recently it has been suggested that the term “diabetes-associated cognitive decline” (DACD) describes a state of mild to moderate cognitive impairment, in particular psychomotor slowing and reduced mental flexibility, not attributable to other causes [5]. In addition, it is now clear that diabetes increases the risk of Alzheimer's disease, vascular dementia, and any other type of dementia [6, 7].

## 2. Pathophysiological Mechanisms Involved in Brain Damage in Diabetes

Long-term effects of diabetes on the brain are manifested at structural, neurophysiological, and neuropsychological level, and multiple pathogenic factors appear to be involved in the pathogenesis of the cerebral dysfunctioning in diabetes, such as the hypoglycemic episodes, cerebrovascular alterations, the role of insulin in the brain, and the mechanisms of hyperglycemia induced damage [8]. Moreover, the emerging view is that the diabetic brain features many symptoms that are best described as accelerated brain ageing [9].

A common theory, for aging and for the pathogenesis of this cerebral dysfunctioning in diabetes, relates cell death to oxidative stress mediated by free radicals [10]. Thus, hyperglycemia reduces antioxidant levels and concomitantly increases the production of free radicals. These effects contribute to tissue damage in diabetes mellitus, leading to alterations in the redox potential of the cell with subsequent activation of redox-sensitive genes [11].

The brain is especially vulnerable to oxidative damage as a result of its high oxygen consumption rate, abundant lipid content, and relative paucity of antioxidant enzymes as compared to other tissues. Neuronal cells are particularly sensitive to oxidative insults, and therefore reactive oxygen species (ROS) are involved in many neurodegenerative processes such as diabetes [12–14]. Although under normal physiological conditions a balance exists between the production of ROS and the antioxidant mechanisms, it has been shown that in aging tissues oxidative stress increases due to, among others, decreased activity of antioxidant enzymes [15]. Earlier work and ample evidence have shown that peroxidative damage to lipid and protein occurs with the aging process and the products of these reactions accumulate in the brain with age [16–19].

Similarly, the activities of superoxide dismutase and catalase or glutathione peroxidase enzymes, involved in the antioxidant defense of the diabetic brain, are decreased [20–23]. However, the possible source of oxidative stress in brain injury also includes autoxidation of glucose, lipid peroxidation, and decreased tissue concentrations of low molecular weight antioxidants such as reduced glutathione (GSH) [24–27]. This alteration of glutathione levels may be related to an increased polyol pathway [28] activity as this leads to a depletion of NADPH which is necessary for the enzymatic reduction of oxidized glutathione.

Moreover, in these pathological conditions, cellular stress triggers mitochondrial oxidative damage, which may result in apoptosis and/or necrosis [29], and apoptosis induced by oxidative stress has been related to neurogenesis inhibition [30]. Thus, it has been described that DM leads to alterations in the mitochondrial electron transport chain; ROS formation, mitochondrial energy metabolism dysfunction, and oxidative stress are thus being recognized as the main players in diabetes-related complications [31]. In this sense, Cardoso et al. have shown that hippocampal mitochondria of streptozotocin (STZ)-induced diabetic rats presented higher levels of MDA together with an increased glutathione disulfide reductase activity and lower manganese superoxide dismutase (MnSOD) activity and glutathione-to-glutathione disulfide (GSH/GSSG) ratio. It also showed impaired oxidative phosphorylation system characterized by a decreased mitochondrial energization potential and ATP levels and higher repolarization lag phase [32]. On the other hand, although insulin is best known for its involvement in the regulation of glucose metabolism in peripheral tissues, this hormone also affects numerous brain functions including cognition, memory, and synaptic plasticity through complex insulin/insulin receptor (IR) signaling pathways [33]. Therefore, considering the important role of insulin in many aspects of neuronal function in both the peripheral nervous system and the central nervous system, it is possible that perturbation of insulin signaling (both insulin deficiency in T1 diabetes and hyperinsulinemia in T2 diabetes) is in the pathogenesis of neurological diseases [34] and results in neurodegeneration.

Until recently, the study of insulin resistance was mainly focused on metabolic tissues such as muscle and adipose tissue; recent data, however, suggest that insulin resistance also develops in the nervous system. Although neurons are not insulin-dependent, they are insulin-responsive [35]. Insulin receptors are widely expressed in the brain, including the olfactory bulb, cerebral cortex, hippocampus, hypothalamus, and amygdala. Insulin resistance in sensory neurons makes cells respond inappropriately to growth factor signals, and this impairment may contribute to the development of neurodegeneration and subsequent diabetic neuropathy. Moreover, insulin regulates mitochondrial metabolism and oxidative capacity through PI3K/Akt signaling [36, 37]; therefore, decreased Akt signaling by hyperinsulinemia- mediated IR may have profound effects on mitochondrial function in neurons and result in subsequent increased oxidative stress [38]. In fact, two of the leading theories that have emerged to explain insulin resistance center on mitochondrial function/dysfunction, although interestingly with opposite views. In one theory, inherited or acquired mitochondrial dysfunction is thought to cause an accumulation of intramyocellular lipids that lead to insulin resistance and implies that strategies to accelerate flux through *β*-oxidation should improve insulin sensitivity [39]. In the second theory, the impact of cellular metabolic imbalance is viewed in the context of cellular and mitochondrial bioenergetics, positing that excess fuel relative to demand increases mitochondrial oxidant production and emission, ultimately leading to the development of insulin resistance. In this case, elevated flux via *β*-oxidation in the absence of added demand is viewed as an underlying cause of the disease. Therefore, mitochondrial-derived oxidative stress is fairly well established as an underlying mechanism responsible for the pathological complications associated with diabetes [40], but it also has a role as a primary factor in the development of insulin resistance (and subsequent overt diabetes), since strong experimental evidence from various animal models utilizing mitochondrial targeted approaches has established a link between mitochondrial-derived ROS and insulin resistance in vivo [41, 42].

In conclusion, convincing evidence is now available from previous studies to prove the role of oxidative stress in the development of neuronal injury in the diabetic brain and the beneficial effects of antioxidants. More concretely, the beneficial effect of lutein and DHA in the brain of diabetic animals and the way that these substances were able to ameliorate the oxidative stress present in diabetes has been studied by our group [27, 43]. However, we must take into account, that there are also studies which report the lack of effect of antioxidants in diabetic complications. Thus, Je et al. [44] reported that vitamin C supplementation alone shows limited therapeutic benefit in type 1 diabetes and is more commonly used in combination with vitamin E or other agents [44]. Moreover, most of the evidences favoring the increased oxidative stress in diabetes come from studies in experimental models of diabetes in which the degree of hyperglycemia is excessive. Supportive evidence is also available in studies of human subjects with diabetes; however interventional studies using select antioxidant supplements have failed to show significant benefits of supplementation, as reviewed by Hasanain and Mooradian [45]. The completion of some of the ongoing large clinical trials will shed additional light on the clinical merit of antioxidant supplementation.

## 3. Inflammation in Diabetes

Inflammation represents a fundamental biological process which stands as the foreground of a large number of acute and chronic pathological conditions, and this occurs in response to any alteration of tissue integrity in order to restore tissue homeostasis through the induction of various repair mechanisms. Proper regulation of these mechanisms is essential to prevent uncontrolled amplification of the initial inflammatory response and shift from tissue repair towards collateral damage and disease development [46].

The appropriate recognition of the danger by the host is primordial for the elaboration of proper adaptive responses. Sensing of pathogen-associated molecular patterns (PAMPs) and damage-associated molecular patterns (DAMPs) is ensured by a complex set-up of pattern-recognition' receptors (PRRs), which include, among others, the receptor for advanced glycation end-products (RAGE). PRR activation triggers a wealth of intracellular signaling pathways, including kinases (e.g., MAP kinases, PI3 kinase), adaptors, transcription factors (mainly nuclear factor-*κ*B (NF*κ*B)), and activator protein-1. Such signaling cascades foster the expression of cytokines, chemokines, enzymes, growth factors, and additional molecules that are required for tissue repair [47] and homeostasis restoration. However, there are situations in which such restoration may not adequately occur, resulting in persistent cellular stress, perpetuating and amplifying the inflammatory response. In these conditions, the process leads to significant alterations of tissue functions, with systemic and persistent derangements of homeostasis [48]. Diabetes and neurodegenerative diseases are typical examples of these pathological processes associated with such chronic inflammatory changes [49].

The release of reactive oxygen species has long been recognized as a typical consequence of immune cell stimulation [50, 51], and both acute and chronic inflammatory states are coupled with significant alterations of redox equilibrium, due to the associated enhancement of oxidant generation [49, 52–54]. Accordingly, mitigating oxidative stress by the use of antioxidants has been evaluated as a potentially useful anti-inflammatory strategy in such conditions, as recently reviewed [55]. Overall, the results of innumerable studies have clearly pointed out the strong association between oxidative stress and inflammation. Since responses triggered by Toll-like receptors (TLRs) are conveyed primarily by the activation of NF*κ*B, which is a master regulator of inflammation, controlling the expression of hundreds of genes implicated in innate immune responses, and also a redox sensitive nuclear factor involved in the control of a large number of normal cellular and tissue processes, NF*κ*B has a strategic position at the crossroad between oxidative stress and inflammation.

NF*κ*B transcription factors are ubiquitously expressed in mammalian cells. These proteins are highly conserved across species, and in mammals the NF*κ*B family (also known as the Rel family) consists of five members: p50, p52, p65 (also known as RelA), c-Rel, and RelB. Rel family members function as dimers and the five subunits can homodimerize or heterodimerize. All family members share a Rel homology domain, which contains the crucial functional regions for DNA binding, dimerization, nuclear localization, and interactions with the I*κ*B inhibitory proteins. NF*κ*B dimers exist in a latent form in the cytoplasm bound by the I*κ*B inhibitory proteins, and when NF*κ*B-inducing stimuli activate the I*κ*B kinase complex that phosphorylates I*κ*B, this leads to its ubiquitination and subsequent degradation in the canonical NF*κ*B activation pathway. I*κ*B degradation exposes the DNA-binding domain and nuclear localization sequence of NF*κ*B and permits its stable translocation to the nucleus and the regulation of target genes [56]. Thus, activated NF*κ*B enters the nucleus to induce transcription of a myriad of genes that mediate diverse cellular processes such as immunity, inflammation, proliferation, apoptosis, and cellular senescence [57].

Together with the evidences that relate oxidative stress and inflammation to the pathophysiology of diabetes, studies performed in a variety of cell and animal based experimental systems also suggest that NF*κ*B activation is a key event early in the pathobiology of this disease and its complications [27, 58, 59]. In fact, several studies have highlighted the activation of NF*κ*B by hyperglycemia and its relationship with diabetic complications, as reviewed by Patel and Santani in 2009 [59]; thus, hyperglycemia triggers a number of mechanisms that are thought to underlie diabetic neuropathy. Studies in different experimental models have established that neuronal dysfunction is closely associated with the activation of NF*κ*B and the expression of proinflammatory cytokines [60, 61]. Moreover, NF*κ*B pathway has been revealed as a key molecular system involved in pathological brain inflammation [62], and also experimental studies [52] have suggested that neuronal apoptosis, which is related to NF*κ*B activation, may play an important role in neuronal loss and impaired cognitive function. Additionally, in the hippocampus of streptozotocin-treated rats, not only a strong increase in oxygen reactive species is observed but also a persistent activation of NF*κ*B is observed [23, 27]. Activated NF*κ*B can induce cytotoxic products that exacerbate inflammation and oxidative stress and promote apoptosis [63], leading to oxidative stress induced cell dysfunction or cell death, respectively [64]. However, it should not be forgotten that although NF*κ*B is widely known for its ubiquitous roles in inflammation and immune responses and in control of cell division and apoptosis (and these roles are apparent in the nervous system), neurons and their neighboring cells employ the NF*κ*B pathway for distinctive functions as well, ranging from the development to the coordination of cellular responses to injury of the nervous system and to brain-specific processes such as the synaptic signaling that underlies learning and memory [60]. Therefore, understanding the function of NF*κ*B transcription factors in the nervous system is now a new frontier for the general field of NF*κ*B research, for the investigation of transcriptional regulation in complex neuronal systems, and for the understanding of pathological mechanisms of neurodegenerative diseases.

On the other hand, we cannot forget that type 2 (T2D) diabetes is an overnutrition related disease which usually is preceded by the metabolic syndrome, a common metabolic disorder that results from the increasing prevalence of obesity which includes several interconnected abnormalities such as insulin resistance, impaired glucose tolerance, dyslipidemia, and high blood pressure [65]. Moreover, overnutrition is considered as an independent environmental factor that is targeted by innate immune system to trigger an atypical form of inflammation, which leads to metabolic dysfunctions among others, in the central nervous system (CNS) and particularly in the hypothalamus [62, 66–69], which indeed is known to govern several metabolic functions of the body including appetite control, energy expenditure, carbohydrate and lipid metabolism, and blood pressure homeostasis [70, 71].

**Figure 1:**
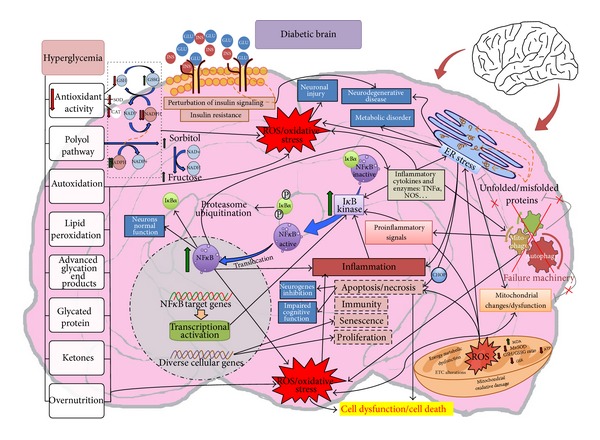
Scheme summarizing the involvement of oxidative stress (mitochondrial dysfunction and ER stress), inflammation, and autophagy in the diabetic brain. GSH: reduced glutathione; GSSG: glutathione disulfide; SOD: superoxide dismutase; NADP^+^: nicotinamide adenine dinucleotide phosphate oxidized; NADPH: nicotinamide adenine dinucleotide phosphate reduced; NAD^+^: nicotinamide adenine dinucleotide oxidized; NADH: nicotinamide adenine dinucleotide reduced; CAT: catalase; I*κ*Ba: nuclear factor of kappa light polypeptide gene enhancer in B cells inhibitor, alpha; NF*κ*B: nuclear factor kappa-light-chain-enhancer of activated B cells; ER: endoplasmic reticulum; GLU: glucose; INS: insulin; P: phosphate; MDA: malondialdehyde; ATP: adenosine triphosphate; ETC: electron transport chain; ROS: reactive oxygen species; MnSOD: manganese superoxide dismutase; GSR: glutathione reductase; CHOP: C/EBP Homology Protein; TNF*α*: tumor necrosis factor alpha; NOS: nitric oxide synthases.

Deeping into the mechanisms that lead to this metabolic dysfunction, which also affects the CNS, it has been recently demonstrated that the activation of IKK*β*/NF*κ*B and consequently the proinflammatory pathway are a relevant feature in different metabolic disorders related to overnutrition [72–74]. The effects of NF*κ*B-mediated metabolic inflammation are deleterious and can give rise to impairments of normal intracellular signaling and disruptions of metabolic physiology [62] that have been reported also in the CNS—particularly in the hypothalamus—which primarily could account for the development of overnutrition-induced metabolic syndrome and related disorders such as obesity, insulin resistance, T2D, and obesity-related hypertension [68, 75, 76]. Moreover, intracellular oxidative stress and mitochondrial dysfunction seem to be upstream events that mediate hypothalamic NF*κ*B activation under overnutrition, and in turn such metabolic inflammation is reciprocally related to the induction of various intracellular stresses such as mitochondrial oxidative stress and endoplasmic reticulum (ER) stress [62]. Thus, intracellular oxidative stress seems to contribute to metabolic syndrome and related diseases, including T2D [39, 77, 78], and also to neurodegenerative diseases [79, 80]. In fact, when ROS homeostasis is disrupted, excessive ROS are accumulated in the mitochondria and cytoplasm and can cause oxidative damage to cells [81]. Regarding the ER, existing evidence also suggests that ER stress is a key link to obesity, insulin resistance, and type 2 diabetes [82], since this ER stress can also activate cellular inflammatory pathways which, in turn, impair cellular functions and lead to metabolic disorders [83] and neurodegenerative diseases [84, 85]. Indeed, unresolved ER stress can induce mitochondrial changes and finally cell apoptosis [86]. Moreover, brain ER stress is known to promote NF-*κ*B activation in the development of central metabolic dysregulations associated to inflammatory pathways, since intraventricular infusion of an ER stress inhibitor suppressed the activation of hypothalamic NF*κ*B by high-fat diet feeding [68]. In addition, ER stress also appears to depend on IKK*β*/NF*κ*B pathway activity, because neither high-fat diet feeding nor central administration of chemical ER stress inducer is able to induce hypothalamic ER stress in mice with central inhibition of IKK*β*/NF*κ*B pathway [68, 87]. Finally, ER stress also causes cellular accumulation of ROS associated to oxidative stress [88], which in turn reciprocally can promote ER stress (see Figure 1).

In the case of ER stress, exposure to high glucose could induce ER stress by the generation of free radicals, aberrant protein glycosylation, or increased membrane and protein turnover. Zhang et al. have also reported that the expression of C/EBP homology protein (CHOP), the prominent mediator of the ER stress-induced apoptosis, was markedly increased in the hippocampus of diabetic rats and have suggested that this CHOP- ER stress-mediated apoptosis may be involved in hyperglycemia-induced hippocampal synapses and neuronal impairment and promote the diabetic cognitive impairment [89].

## 4. Autophagy and Diabetes

Autophagy plays a role in the maintenance of function of organelles such as mitochondria or ER [90, 91], in order to maintain a healthy and functional intracellular environment, cells must constantly clean up defective proteins (e.g., misfolded proteins overflowing from ER stress) or damaged organelles (e.g., dysfunctional mitochondria or ER from prolonged oxidative stress). Although, autophagy is known primarily as a prosurvival mechanism for cells facing stress conditions, accumulating evidence indicates that autophagy can contribute to cell death processes under pathological conditions [92, 93]. Thus, among others, autophagy defect has been linked to the development of metabolic syndrome, diabetes, alcoholism, and lipid abnormalities [94–96], and in the majority of these cases, the underlying pathogenesis is related to the failure of autophagy machinery to efficiently remove defective proteins or damaged organelles from the cytosol. In fact, chronic intracellular stress such as mitochondria or ER stress seems to be the critical upstream events, since animal studies have shown that in early stages ER stress or oxidative stress induce adaptive autophagy upregulation, helping to restore intracellular homeostasis by disposing a number of harmful molecules such as unfolded or misfolded proteins in ER lumen, cytosolic proteins damaged by ROS, or even dysfunctional ERs and mitochondria [97, 98]. However, when intracellular stresses remain unresolved, prolonged autophagy upregulation progresses into autophagy defect [62] and, in fact, the decreased efficiency of the autophagic system with age has gained renewed attention as a result of the increasing number of reports supporting a role for defective autophagy in the pathogenesis of different age-related diseases including diabetes among others [99]. In parallel, autophagy pathway can relate to proinflammatory signaling via oxidative stress pathway [100], since mitophagy/autophagy blockade leads to the accumulation of damaged, ROS-generating mitochondria, and this in turn activates the NLRP3 inflammasome (a molecular platform activated upon signs of cellular “danger” to trigger innate immune defenses through the maturation of proinflammatory cytokines). Moreover, autophagy defect can induce NF*κ*B-mediated inflammation [101, 102], even in the CNS, since Meng and Cai reported that defective hypothalamic autophagy led to hypothalamic inflammation, including the activation of proinflammatory I*κ*B kinase *β* pathway [103].

Although it is clear that diabetes affects both mitochondria and ER, the role of autophagy in diabetes or metabolism is yet far from clear, and therefore the role of autophagy in the pathogenesis of diabetic complications is currently under intensive investigation.

As described by Hoffman et al., [104] specific candidates for induction and stimulation of autophagy include insulin deficiency/resistance [105, 106]; deficiency of insulin growth factor-1 (IGF-1) and insulin growth factor-1 receptor (IGF-1R) [104, 107]; hyperglucagonemia [106]; and hyperglycemia [107]. Other candidates for perturbation of autophagy include alteration of protein synthesis and degradation [108] due to the oxidative stress of RNA [109, 110], protein damage, and altered lipid metabolism [94, 111]; increased production of ketones and aldehydes [112, 113]; and lipid peroxidation [110, 114]. Furthermore, accumulation of oxidized and glycated proteins, common protein modifications associated with diabetes, could be in part attributed to defective autophagy [115].

It is noteworthy that Hoffman et al. have reported that autophagy is increased in the brains of young T1D patients with chronic poor metabolic control and increased oxidative stress [116]. Moreover, the finding of significant expression of autophagic markers in both white and gray matter is in keeping with the structural deficits in young patients with T1D [117, 118] and the white matter atrophy in the frontal and temporal regions in these diabetic ketoacidosis cases [104]. However there are still few studies focusing on the role of autophagy in the brains of T1D patients, and therefore further research is needed on the relationship between autophagy and pathogenesis of early onset diabetic encephalopathy in T1D.
